# Whole plant acclimation responses by finger millet to low nitrogen stress

**DOI:** 10.3389/fpls.2015.00652

**Published:** 2015-08-19

**Authors:** Travis L. Goron, Vijay K. Bhosekar, Charles R. Shearer, Sophia Watts, Manish N. Raizada

**Affiliations:** Department of Plant Agriculture, University of GuelphGuelph, ON, Canada

**Keywords:** finger millet, nitrogen stress, grain, shoot, root, root hair, crown root, lateral root

## Abstract

The small grain cereal, finger millet (FM, *Eleusine coracana* L. Gaertn), is valued by subsistence farmers in India and East Africa as a low-input crop. It is reported by farmers to require no added nitrogen (N), or only residual N, to produce grain. Exact mechanisms underlying the acclimation responses of FM to low N are largely unknown, both above and below ground. In particular, the responses of FM roots and root hairs to N or any other nutrient have not previously been reported. Given its low N requirement, FM also provides a rare opportunity to study long-term responses to N starvation in a cereal species. The objective of this study was to survey the shoot and root morphometric responses of FM, including root hairs, to low N stress. Plants were grown in pails in a semi-hydroponic system on clay containing extremely low background N, supplemented with N or no N. To our surprise, plants grown without deliberately added N grew to maturity, looked relatively normal and produced healthy seed heads. Plants responded to the low N treatment by decreasing shoot, root, and seed head biomass. These declines under low N were associated with decreased shoot tiller number, crown root number, total crown root length and total lateral root length, but with no consistent changes in root hair traits. Changes in tiller and crown root number appeared to coordinate the above and below ground acclimation responses to N. We discuss the remarkable ability of FM to grow to maturity without deliberately added N. The results suggest that FM should be further explored to understand this trait. Our observations are consistent with indigenous knowledge from subsistence farmers in Africa and Asia, where it is reported that this crop can survive extreme environments.

## Introduction

Finger millet (FM, *Eleusine coracana* L. Gaertn) is one of the small millet cereals (National Research Council, 1996; Goron and Raizada, 2015), originally native to the Ethiopian highlands (Dida et al., 2008). FM is largely consumed by marginalized inhabitants of semi-arid Asia and Africa, and sold to provide subsistence farmers with additional income (Dorosh et al., 2009; Gruère et al., 2009). FM is also highly valued by local farmers for its ability to grow in adverse agro-climatic conditions, where major cereal crops such as maize (*Zea mays*), wheat (*Triticum* spp.), and rice (*Oryza sativa*) fail, and has been noted to tolerate a wide variety of soils (Upadhyaya et al., 2006).

Related to the latter point, FM is also valued for its exceptionally high nitrogen use efficiency (NUE; Gupta et al., 2014), defined as grain yield per unit of available nitrogen (N; Moll et al., 1982). Compared to other grain crops such as maize, FM responds very well to low amounts of N (Roy et al., 2001). Many subsistence farmers in South Asia have reported to us that FM can grow without any added N or with only residual N. A more formal study carried out by the All India Coordinated Small Millet Improvement Project (AICSMIP) indicated that while FM responds well to urea N application at 90 kg/ha, the cost benefit ratio was highest between 0 and 30 kg N/ha^1^. This is a significantly lower N requirement than corn, for which optimum application rates can be greater than 200 kg/ha (Debruin and Butzen, 2014). Other work has confirmed these observations: a four year study in 2011 showed that FM grain yield stayed consistent between fertilizer application rates of 20–40 kg N/ha (Pradhan et al., 2011). Limited research also suggests that FM genotypes vary in their NUE (Thilakarathna and Raizada, 2015), with some genotypes recognized as having higher responsiveness to applied N (Puttaswamy and Krishnamurthy, 1976; Bhoite and Nimbalkar, 1996; Dubley and Shrivas, 1999; Gupta et al., 2012, 2013, 2014). There is paucity of literature concerning development of new FM genotypes with high yield potential under low N application regimes (Thilakarathna and Raizada, 2015). However, in recent years more work has been accomplished in this area (Goron and Raizada, 2015). Breeding efforts can be facilitated by selection for traits associated with NUE.

Despite FM being recognized as a high NUE crop (Gupta et al., 2014), the underlying mechanisms are not well understood. Crop plants utilize a wide range of acclimation strategies to mitigate the limitations of low N availability, both morphological and biochemical, including altering root traits for better nutrient salvaging, decreasing chlorophyll production, changing N allocation within the plant, and altering the timing of flowering (Chapin et al., 1987; Ciampitti et al., 2013).

**FIGURE 1:**
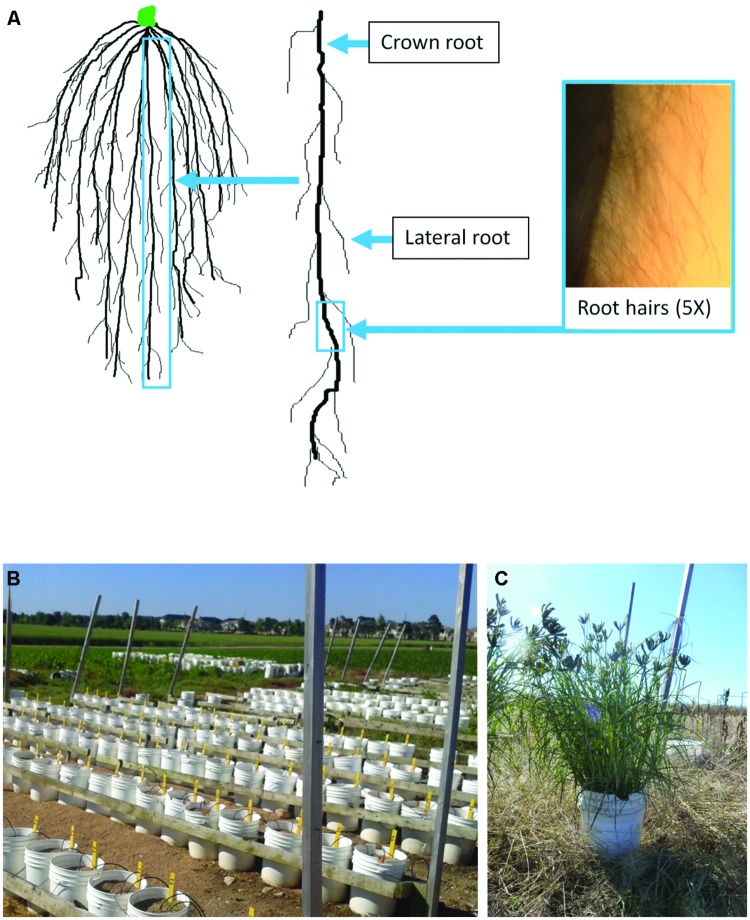
**Guide to the FM root system and the semi-hydroponic field fertigation growth system used in this study.** Diagram of a FM root network **(A)**. Crown roots are the thick roots that initiate from the crown region at the base of the shoot. Lateral roots initiate from the crown roots and can subsequently branch. Root hairs are present on both crown roots (as shown) and lateral roots. The fertigation growth system employed in this study consisted of 22 L plastic pails, 28 cm in diameter, filled with an inert baked clay medium (Turface^®^ ; **B**). Plants were allowed to grow to maturity as shown **(C)**. Irrigation hoses delivered the nutrient solution except for nitrogen which was added manually as described.

Particularly poorly characterized acclimation responses to low N in plants include changes in root growth and architecture. The cereal root system consists of thick crown roots which initiate from the base of the stem (the crown region), from which lateral roots extend and branch; all of these root types can further initiate root hairs, which are epidermal projections that assist with nutrient uptake (**Figure 1A**). Maize exhibits diverse root responses to low N, for example by increasing the total length of the root system, decreasing the crown root number, and increasing the lateral root to crown root ratio (Eghball and Maranville, 1993; Wang et al., 2004; Chun et al., 2005; Liu et al., 2009; Gaudin et al., 2011a). To a lesser extent, low N is associated with altered root hair length and density in crop grasses (Gaudin et al., 2011a,b). Under extreme N deficiency (N starvation), *Arabidopsis thaliana* ecotypes show a range of root responses to N starvation (Ikram et al., 2012), though such extreme experiments are more difficult to conduct with the major cereals, because of their high N requirement for viability after the seedling stage.

To the best of our knowledge, there have been no reports concerning root architecture or root hair traits in FM except for a recent root hair methodology paper by our group (Goron et al., 2015). Furthermore, we could not find reports in the literature concerning other detailed morphological acclimation responses of FM in response to low N. Given its low N requirement and small seed size as a source of starter N, FM may also provide a rare opportunity to understand long-term acclimation responses of a cereal to extreme N stress.

The objective of this study was to survey shoot and root morphometric acclimation responses of FM to very low background N. To ensure minimal levels of N, plants were grown in pails containing an inert clay substrate called Turface^®^ in a semi-hydroponic system without added N (Tollenaar and Migus, 1984; **Figures 1B,C**). This system permitted a more detailed analysis of fine root traits including root hairs, as shown by our group with maize (Gaudin et al., 2011a,b) and recently in FM (Goron et al., 2015), compared to excavation from soil. At the beginning of the study, it was unclear whether FM plants would reach maturity in the absence of added N.

## Materials and Methods

### Plant Materials and Growth Conditions

A field experiment was carried out over two growing seasons (2012 and 2013). A commercial variety of *Eleusine coracana* L. Gaertn (FM) seeds was obtained from India. Seeds were germinated in open trays at room temperature, supplied with only double distilled water (ddH_2_O) in a laboratory for 7 days before transplantation into pails in a field near Guelph, Canada (43°53′N, 80°18′W, 325 m above sea level). Single FM plants were grown in 22 L plastic pails (28 cm in diameter) containing Turface^®^ MVP (Profile Products LLC., Buffalo Grove, IL, USA). This is an inert, baked-clay, coarse growth medium which can be used in automated field fertigation systems as previously described by Tollenaar and Migus (1984). Within the field, the distance between the centers of pails in each row was 35 cm, and the spacing between rows was 142 cm. Pails were arranged in blocks consisting of two N treatments (see below). There were four blocks per row, with blocks distributed in the field across five rows, for a total of 20 replicates per treatment per season. Each block was flanked by buffer plants which received varying amounts of N. Plants were sampled randomly.

Millet plants were irrigated by an automated mechanism (**Figure 1B**), zero to three times per day, adjusted throughout the growing season as required. A concentrated, modified Hoagland’s solution lacking N was stored in a 340 L reservoir at the field site, and was diluted to the appropriate concentration by the irrigation mechanism at a ratio of 1:100 during application. The pH of the diluted solution was adjusted to 6.5–6.7 by the addition of HCl. Two fertigation tubes calibrated to deliver a minimum of 10 ml of nutrient solution per minute were inserted into each pail (Page et al., 2011). For the positive nitrogen treatment (+N), 1.1 g of urea was dissolved into 1 L H_2_0 (37 mM total N, to compensate for leaching) and was provided to the plants at weekly intervals (13 times total) over the course of the experiment, with control plants receiving 1 L H_2_O with no nitrogen (-N) per dose. Nitrogen (or the water control) was supplied directly to each experimental unit by hand at the same time of day throughout the experiment.

### Physiological, Seed Head, Shoot, and Root System Measurements

Chlorophyll levels of shoot tissue from a minimum of five randomly selected plants in each treatment were obtained with a Konica Minolta SPAD-502Plus chlorophyll meter (Konica Minolta, Inc., Tokyo). Similar leaf positions were sampled between treatments. In 2012, one measurement was made at 106 DAT; in 2013 two measurements were made at 50 and 80 DAT. In both years, plant flowering was recorded and tracked over time. A plant was considered to have flowered on the day at which the inflorescence was first visible. Tiller number was scored at 113 DAT in 2012 and 119 DAT in 2013.

Seed head tissues were harvested for biomass analysis at 134 DAT in 2012 and 135 DAT in 2013. For root and shoot biomass analyses, at least 10 plants per treatment were collected at 142 DAT in 2012 and 139 DAT in 2013. In 2013, three samples of each tissue (10 g each of root, shoot, and seed heads) were dehydrated in a tissue dryer at 82°C, ground to a fine powder in liquid nitrogen, and sent to the University of Guelph Agriculture and Food Laboratory for total N content quantification with the Dumas combustion method (Fiedler et al., 1973) using a LECO FP428 nitrogen and protein determinator (LECO Corp., St Joseph, MI, USA).

At the time of harvest in both years, five complete root systems from each treatment were frozen at -20°C in 50% ethanol. Before analysis, roots were thawed in water and floated in 30 cm × 42 cm transparent plastic trays. Roots were scanned with an Epson Expression 10000XL large area scanner (Seiko Epson Corporation, Suwa, Nagano, Japan), and the resulting images were analyzed with WinRhizo software (Version PRO2009, Regent Instruments Inc., Québec, QC, Canada). The analysis software was set to measure total root length per diameter class allowing separate quantification of lateral roots (<0.45 mm) and crown roots (>0.5 mm). Crown root number was scored by counting visible roots in the crown region.

### Root Hair Microscopy and Measurements

Finger millet root hairs were measured using a protocol recently developed by our group (Goron et al., 2015). Briefly, root systems were thawed by floating them in water. Four distinct crown roots of different lengths were selected from each root system in order to obtain an accurate representation of the range of crown root ages within each root network. The four different crown root length classes were kept constant between plants. Five segments measuring 1 cm in length were dissected from each of the selected crown roots at equally spaced distances along the root, and rinsed in double distilled water. Crown root segments were stained with 0.4% Trypan Blue solution (MP Biomedicals LLC, Solon, OH, USA) for 10 min, and then rinsed five times with ddH_2_O. Root segments were subsequently immersed in 70% glycerol (Sigma-Aldrich, St. Louis, MO, USA), then examined with a Leica MZ8 stereomicroscope (Leica Microsystems GmbH, Wetzlar, Germany) under 5x magnification. Northern Eclipse software (version 5.0, Empix Imaging Inc., Mississauga, ON, Canada) was used to capture four non-overlapping graphic images of each 1 cm root segment with a Sony DXC-950P Power HAD 3CCD color video camera (Tokyo, Japan). ImageJ software (Version 1.47, Wayne Rasband, NIH, USA) was used to manually trace root hairs to quantify root hair length and density by first calibrating the program to the image of a stage micrometer (1 mm in length). For root hair length determination, all four images per crown root segment were used, with up to 10 root hairs in each image traced. For statistical analyses, the mean root hair length from all four images was calculated, representing a pool of up to 40 root hairs. In total, 14,420 root hairs were traced by hand for the experiment. For root hair density determination, for each crown root segment, a randomly selected 300 μm sub-segment was used to count the number of root hairs. For both root hair length and density measurements, there were a total of five replicate plants.

### Determination of Potential Plant-Available Nitrogen in the Clay Growth Medium

A description of the physical-chemical properties of Turface clay is included (Supplementary Table S1). However, as no data was available concerning the amount of N present in our clay gravel growth medium, three replicates of 300 g ground Turface^®^ MVP were sent to the University of Guelph Agriculture and Food Laboratory for total N content quantification, with the Dumas combustion method (Etheridge et al., 1998). The dry clay gravel was found to contain minimal levels of N (0.053%; Supplementary Table S2). Additionally, to determine N bioavailability, 170 g of the growth medium was submerged for 24 h in the same N-free nutrient solution provided to plants in the field. The resulting slurry was filtered to remove the gravel, and three replicates of the filtrate were sent to the same lab listed above for total N quantification by the Kjeldahl method (Keeney and Bremner, 1966). The filtrate was also found to contain extremely low levels of N (1.42 mg/L total N, equivalent to 0.1 mM; Supplementary Table S2).

### Statistical Analysis

Analyses were performed using GraphPad Prism^®^ software (version 6.04; GraphPad Software, Inc., San Diego, CA, USA). ROUT was used to identify and remove outliers at Q = 1%. There was concern for the non-random N treatments of the neighboring border plants, though they were in separate pails. Nevertheless, to compensate for any position effects of neighboring plants on the variance of each treatment, non-parametric Mann–Whitney tests were used to analyze datasets with non-normal values. Non-normality was identified with Shapiro–Wilk tests where replicate numbers were sufficient and with Kolmogorov–Smirnov tests where the sample size was small. Furthermore, unpaired *t*-tests with Welch’s correction for unequal standard deviations were used to generate *P-*values.

Correlation coefficients were calculated by the two-tailed Pearson method with a 95% confidence interval. Hypotheses were evaluated at a type I error rate of 0.10 or 0.05 as indicated.

## Results

### Finger Millet Survival under N Starvation

**FIGURE 2:**
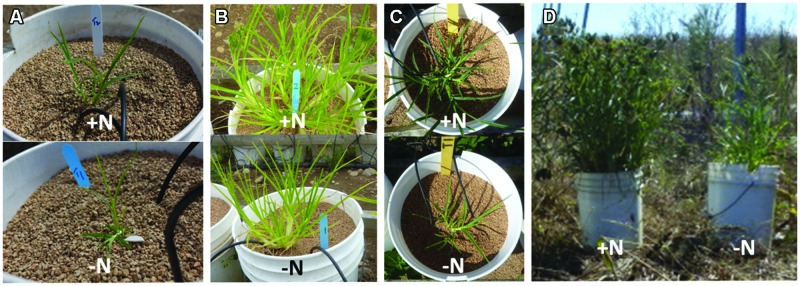
**Finger millet shoot responses to low N at different growth stages.** FM in 2012 at 7 weeks of growth **(A)** and at harvest **(B)**. FM in 2013 at 7 weeks of growth **(C)** and at harvest **(D)**. All pails were 28 cm in diameter.

To our surprise, plants that did not receive nitrogen (-N) deliberately at any point during the experiment (over a ∼140 day period) reached maturity in both 2012 and 2013. The plants looked normal, but smaller and less bushy, compared to plants treated with N (+N), and even produced healthy seed heads (6.5 g per plant in 2012, and 13.7 g per plant in 2013; **Figure 2D**; **Table 1**).

**Table 1:** Measurements of above ground traits of FM in response to low nitrogen stress.

Year	Treatment	Duration	Shoot biomass (g)^∗^	Seed head biomass (g)^#^	Shoot tiller number
2012	+N	142 DAT	92.8 ± 2.23, *n* = 10 A	8.9 ± 0.43, *n* = 10 A	4.0 ± 0.26, *n* = 10 (113 DAT) A
	-N	142 DAT	72.3 ± 0.80, *n* = 10 B	6.5 ± 0.27, *n* = 10 B	2.8 ± 0.20, *n* = 10 (113 DAT) B
2013	+N	139 DAT	869.9 ± 53.83, *n* = 20 a	54.9 ± 6.14, *n* = 20 a	50.5 ± 1.87, *n* = 13 (119 DAT) a
	-N	139 DAT	168.0 ± 13.42, *n* = 20 b	13.7 ± 1.45, *n* = 20 b	19.9 ± 1.51, *n* = 13 (119 DAT) b

*Letters that are different within a year indicate that the means are significantly different (*P* < 0.05).*

*^∗^Dry biomass was measured in 2012, and fresh biomass in 2013.*

*^#^Dry biomass was measured both years.*

### Root, Shoot, and Seed Head Biomass

**FIGURE 3:**
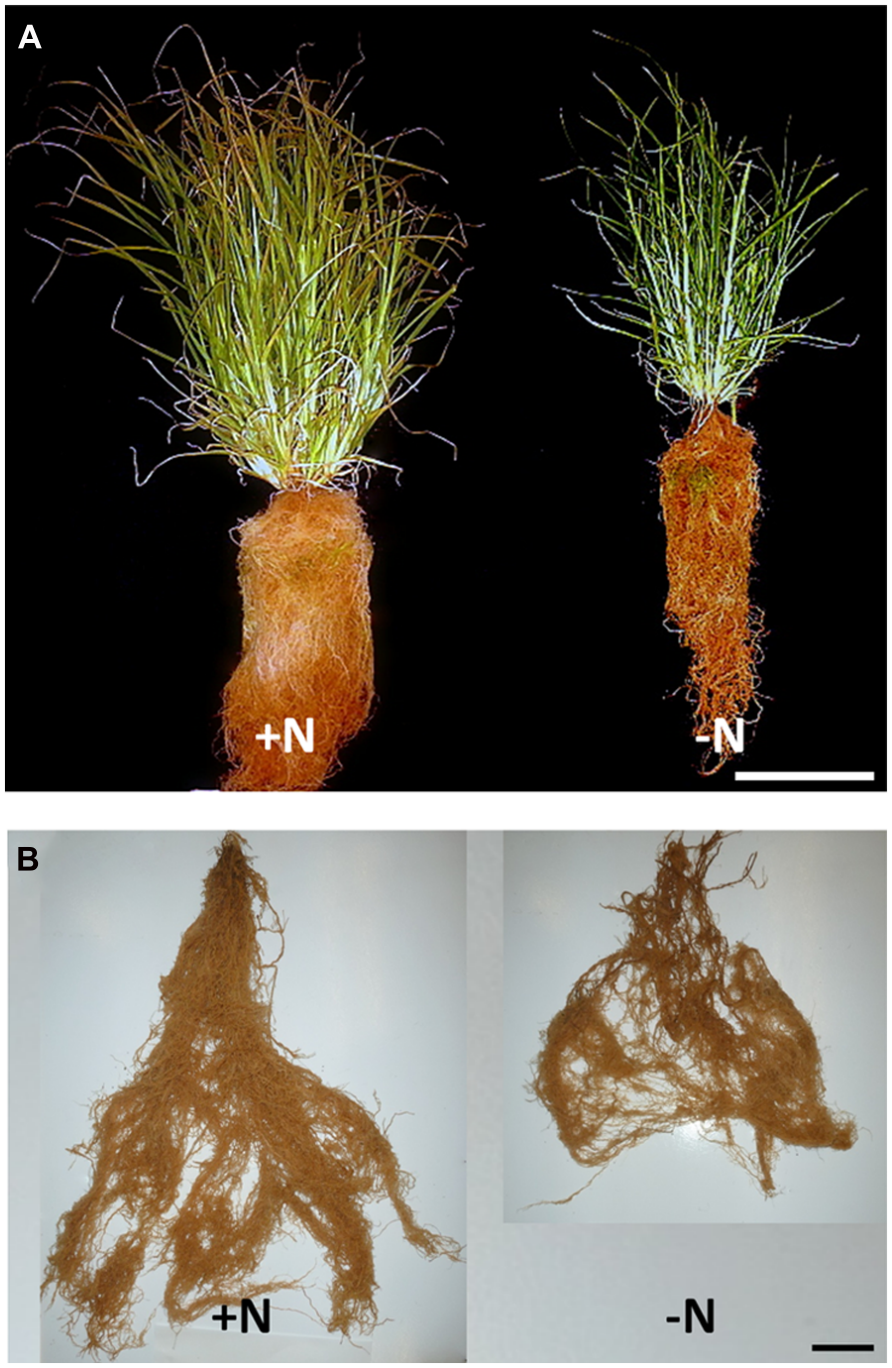
**Shoot and root responses of FM to nitrogen limitation, at harvest.** Representative mature, intact plants harvested in 2013, provided with +N and -N treatments **(A)**. The seed heads were pre-harvested. The white scale bar at the lower right represents a length of 25 cm. Representative mature, entire root networks harvested in 2012 grown under +N and -N treatments, left and right respectively **(B)**. The black scale bar at the lower right represents a length of 10 cm.

In both 2012 and 2013, end-season root and shoot biomass values were significantly lower when plants were treated with -N compared with +N (**Tables 1** and **2**; **Figures 2** and **3**). However, biomass values varied widely between years, with 2012 plants being notably smaller than 2013 plants, a reflection of differences in growing conditions between these years. Due to these differences, as well as differences in methodology (i.e., recording dry shoot biomass in 2012 and fresh shoot biomass in 2013), biomass comparisons were restricted to within each growing season, and no tests of significance across years were attempted. In 2012 and 2013, plants receiving the -N treatment showed 27 and 75% declines in seed head biomass, respectively, compared to plants that had received the +N treatment (**Table 1**).

**Table 2:** Measurements of root traits of FM in response to low nitrogen stress.

Year		Duration	Root biomass (g)^∗^	Crown root number	Total crown root length (mm)	Average crown root length (mm)	Total lateral root length (mm)	Total root length (mm)	Lateral root: crown root ratio
2012	+N	142 DAT	229.9 ± 13.48, *n* = 10 A	80.8 ± 2.65, *n* = 5 A	6490.2 ± 729.71, *n* = 5 A	79.7 ± 6.87, *n* = 5 A	20926.6 ± 1752.15, *n* = 5 A	27415.8 ± 2363.43, *n* = 5 A	3.3 ± 0.20, *n* = 5 A
	-N	142 DAT	56.2 ± 6.07, *n* = 10 B	69.6 ± 7.00, *n* = 5 A	6365.4 ± 1102.39, *n* = 5 A	91.9 ± 13.96, *n* = 5 A	22486.1 ± 2676.98, *n* = 5 A	28851.6 ± 3748.96, *n* = 5 A	3.7 ± 0.25, *n* = 5 A
2013	+N	139 DAT	249.9 ± 37.26, *n* = 15 a	216.5 ± 27.25, *n* = 5 a	11134.1 ± 1124.29, *n* = 4 a	53.2 ± 1.71, *n* = 5 a	54639.1 ± 6949.32, *n* = 4 a	65773.2 ± 7957.09, *n* = 4 a	4.9 ± 0.30, *n* = 4 a
	-N	139 DAT	136.2 ± 26.25, *n* = 13 b	110.8 ± 9.05, *n* = 5 b	6999.8 ± 653.04, *n* = 5 b	64.1 ± 6.53, *n* = 5 a	34576.1 ± 4500.85, *n* = 5 b	41575.9 ± 5123.39, *n* = 5 b	4.9 ± 0.26, *n* = 5 a

*Letters that are different within a year indicate that the means are significantly different (*P* < 0.05). Displayed values are mean±SEM.*

*^∗^Root fresh weight was measured both years.*

### Whole Plant Architecture

Above ground, in both years, the number of tillers per plant decreased significantly when plants were treated with -N compared with +N, with a 30% decrease in 2012 and a 60% decrease in 2013 (**Table 1**). Below ground, complete FM root systems at maturity showed a high degree of fibrousness (**Figure 3**). In 2012, with the smaller plants, no measurements of root architecture aside from biomass differed between N treatments (**Table 2**). In 2013, with the larger, more N-demanding plants, the number of crown roots on -N plants was 49% less than +N plants. Total crown root length, total lateral root length, and total root system lengths were all 37% smaller in -N plants (**Table 2**). There was no difference in terms of average crown root length or the ratio of total lateral to crown root length.

### Correlation between Above Ground Biomass and Root Characteristics

**FIGURE 4:**
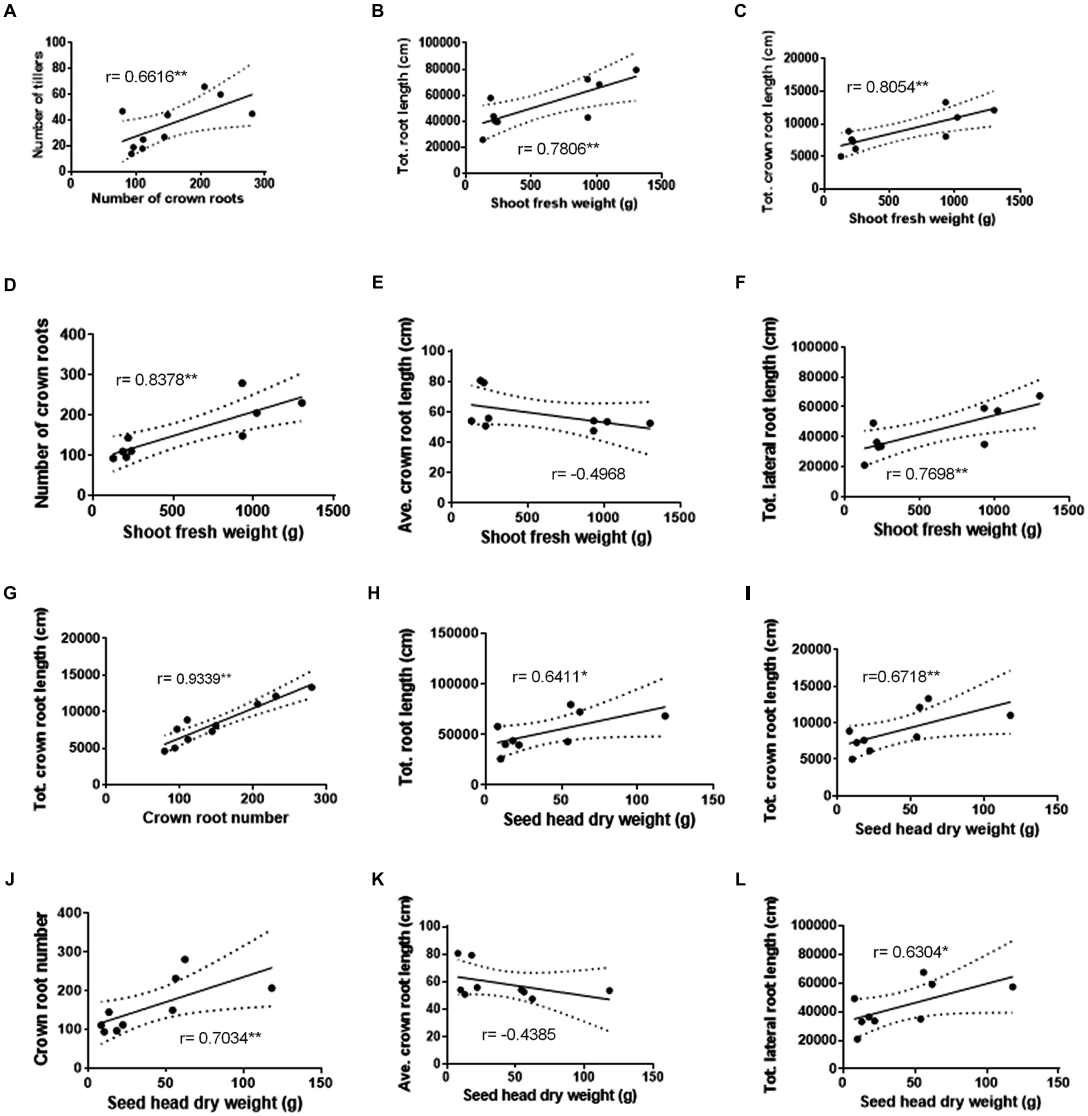
**Correlations between FM shoot and seed head biomass and root architecture traits at harvest across nitrogen treatments.** Number of crown roots vs. tillers **(A)**. Shoot fresh weight vs. total root length **(B)**, shoot fresh weight vs. total crown root length **(C)**, shoot fresh weight vs. number of crown roots **(D)**, shoot fresh weight vs. calculated average crown root length **(E)**, and shoot fresh weight vs. total lateral root length **(F)**. Number of crown roots vs. total crown root length **(G)**. Seed head dry weight vs. total root length **(H)**, seed head dry weight vs. total crown root length **(I)**, seed head dry weight vs. number of crown roots **(J)**, seed head dry weight vs. calculated average crown root length **(K)**, and seed head dry weight vs. total lateral root length **(L)**. Pearson *r* values are displayed. A single asterisk denotes significance at *P* < 0.10. A double asterisk denotes significance at *P* < 0.05.

Pearson correlations were performed between above ground biomass and root characteristics using data collected in 2013 (**Figure 4**; data from individual plants were not available in 2012 to permit correlations). In 2013, a significant correlation was observed between the numbers of crown roots and tillers (**Figure 4A**). The shoot fresh biomass also significantly and positively correlated with the crown root number, total crown root length and total lateral root length (**Figures 4B–D,F**), but not with average crown root length (**Figure 4E**).

Additionally, Pearson correlations were performed between seed head dry biomass and the aforementioned root characteristics for the 2013 season. There were positive correlations between seed head biomass and total root length, crown root number, total crown root length, and total lateral root length (**Figures 4H–J,L**), but not with average crown root length (**Figure 4K**), though some correlations were only significant at *P* = 0.10 (as indicated).

Combined, these data indicate that the declines in shoot and seed head biomass observed under severe low-N stress might be matched by decreases in shoot tiller number as well as the total lengths of the two main root classes.

### Finger Millet Root Hair Quantification

**FIGURE 5:**
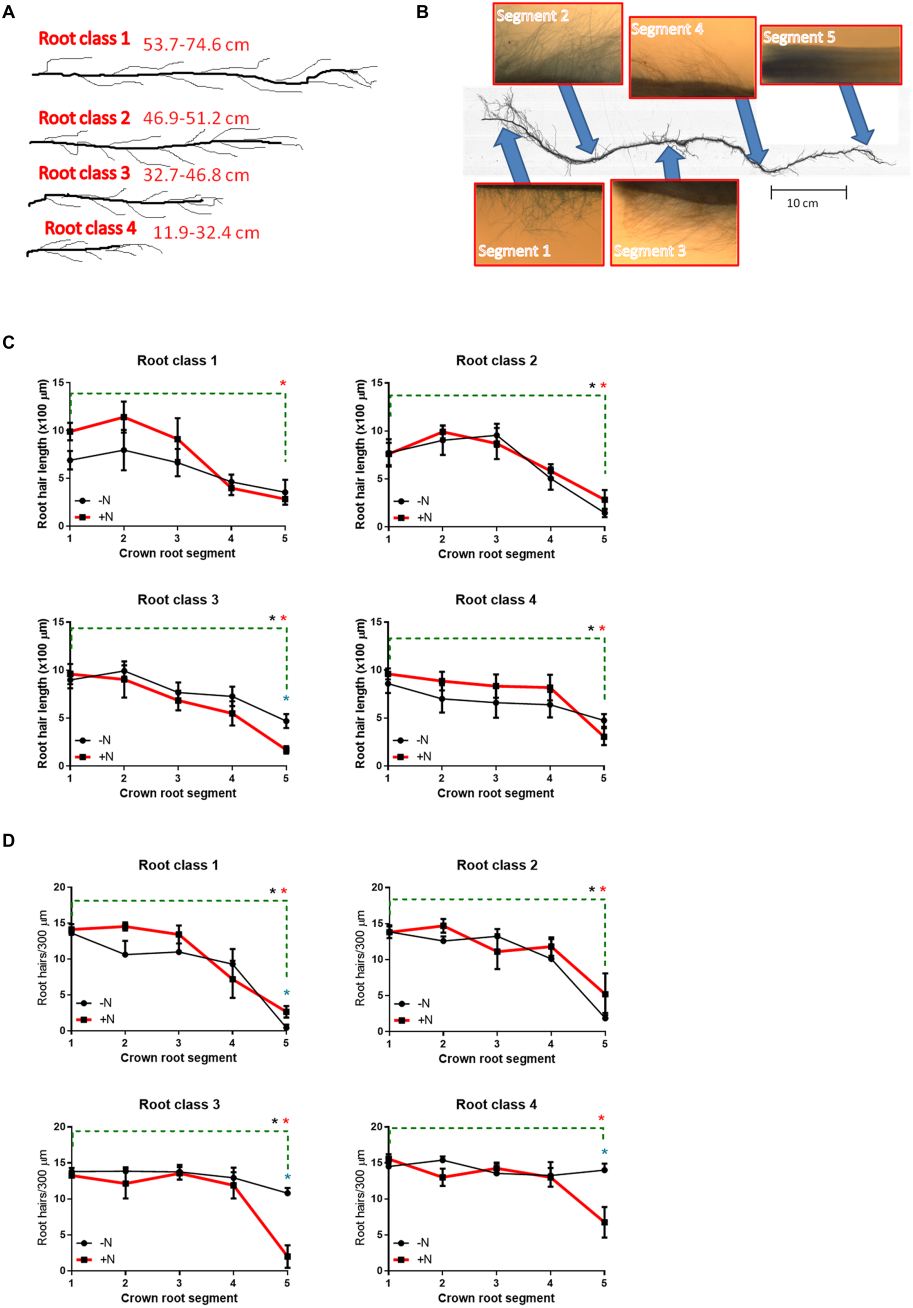
**Root hair morphometric traits from 2013 plants at harvest.** In 2013 and 2012 (data in Supplemental Figure S1), four different classes of crown roots were sampled based on their lengths/ages **(A)**, and then examined for root hairs at five different segments spaced evenly along each crown root using light microscopy with 5x magnification **(B)**. Root hairs were quantified for length **(C)** and density **(D)** at different segments of the crown roots (x-axis) ranging from the top/nearest the shoot (crown root segment 1) to near the root tip (crown root segment 5). A blue asterisk (^∗^) directly above a mean data point denotes a significant difference in the root hair trait (at *P* < 0.05) between N treatments within an individual crown root segment. An asterisk above the green dashed line denotes a significant difference in the root hair trait (at *P* < 0.05) between the top crown root segment (segment 1) and the bottom crown root segment (segment 2) within the +N treatment (red asterisk) or the -N treatment (black asterisk). All statistical analyses were performed with unpaired *t*-tests (with Welch’s correction where unequal variances required).

Root hairs were observed to be widespread along the entire lengths of FM crown roots (**Figures 5C,D**; Supplementary Figure S1). There were no consistent significant differences in root hair length and density in FM in response to N limitation (**Figures 5C,D**; Supplementary Figure S1). However, in some crown root tips, the newly initiating root hairs (on crown root segment 5) were either significantly shorter and/or less dense when N-limited (**Figures 5C,D**; Supplementary Figure S1). Other trends were observed: root hairs were generally longer and more dense in the upper (older) portions of the crown roots, whereas root hairs became shorter and more sparsely distributed toward the tips of the crown roots (**Figures 5C,D**). A similar trend was observed in 2012, though the differences were less pronounced (Supplementary Figure S1).

### Physiological Measurements

In 2013, all plants were examined daily for evidence of flowering, defined as the point at which the emerging flower was first observed. Plants given +N began to flower at 85 DAT, while -N plants did not begin flowering until 89 DAT. In 2012, the FM plants began flowering on the same day in both treatments.

In both 2012 and 2013, leaf chlorophyll was measured. In 2012, the smaller FM plants showed no significant differences in chlorophyll between treatments (**Table 3**). In 2013, with the larger, more N-demanding plants, leaf chlorophyll (at 50 DAT) of -N plants was 56% lower than +N plants. At 80 DAT, the -N treatment had a leaf chlorophyll content that was 23% lower than +N plants (**Table 3**). No significant differences were detected in total N concentration of root, shoot, or seed head tissue (**Table 3**).

**Table 3:** Finger millet tissue chlorophyll and nitrogen content in response to low nitrogen stress.

Year	Measurement	Treatment	
2012	Chlorophyll content (SPAD)	+N	11.3 ± 1.27, *n* = 5 (106 DAT) A
		-N	8.2 ± 1.43, *n* = 5 (106 DAT) A
2013	Chlorophyll content (SPAD)	+N	51.9 ± 4.06, *n* = 20 (80 DAT) a
		-N	39.8 ± 3.07, *n* = 18 (80 DAT) b
2013	Shoot nitrogen content (% dry weight)	+N	1.9 ± 0.06, *n* = 3 (142 DAT) a
		-N	2.1 ± 0.19, *n* = 3 (142 DAT) a
	Root nitrogen content (% dry weight)	+N	1.3 ± 0.31, *n* = 3 (142 DAT) a
		-N	0.9 ± 0.14, *n* = 3 (142 DAT) a
	Seed head nitrogen content (% dry weight)	+N	2.5 ± 0.17, *n* = 3 (142 DAT) a
		-N	2.1 ± 0.17, *n* = 3 (142 DAT) a

*Letters that are different within a year indicate that the means are significantly different (*P* < 0.05).*

## Discussion

Consistent with observations of subsistence farmers, our results show that FM is a remarkable crop – surprisingly able to flower and produce healthy seed heads without any deliberately added nitrogen (N). In our study, plants were grown in an artificial baked clay system with very poor N availability. N-poor environments are typical of the conditions under which this crop is grown in Sub-Saharan Africa and South Asia where no applied N or residual N is used (National Research Council, 1996; Goron and Raizada, 2015). In South Asia, FM is often transplanted from nurseries (National Research Council, 1996), a practice mimicked in this study.

**FIGURE 6:**
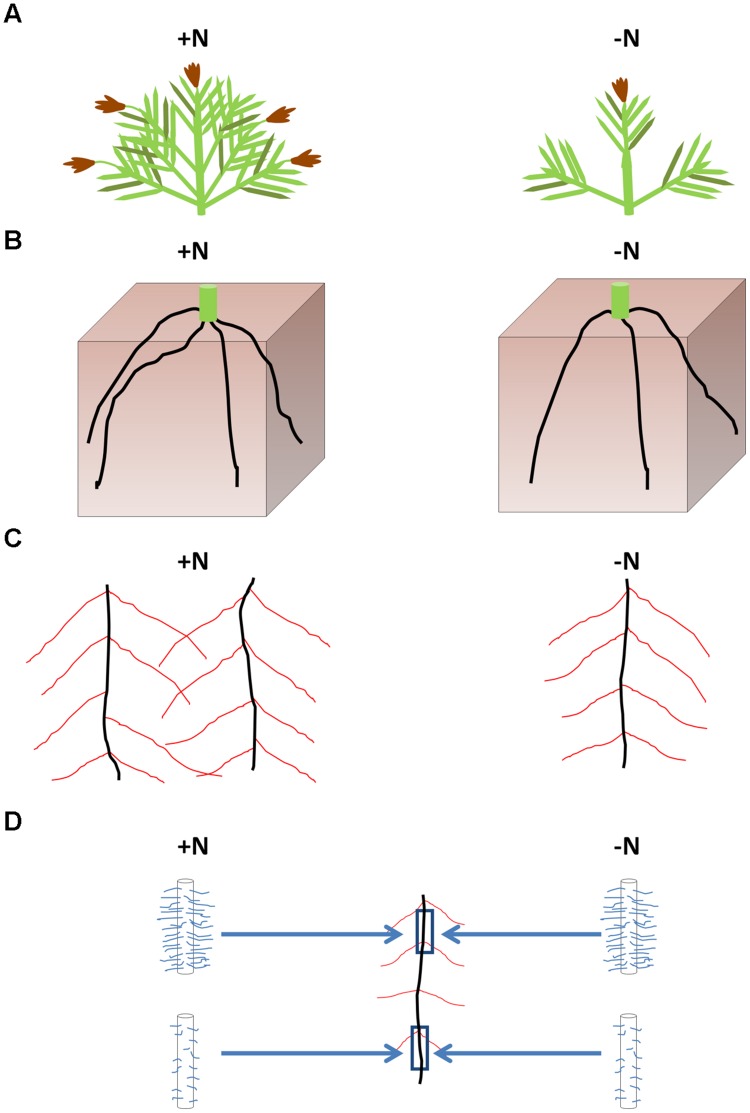
**Summary of the effects of low N stress on FM architecture.** Low N resulted in a reduction in end-season shoot biomass and seed head biomass associated with fewer tillers compared to plants receiving higher N **(A)**. Fewer crown roots were initiated in 2013 in response to the -N treatment **(B)**, likely responsible for the reduction in total lateral root system length **(C)**. The calculated average crown root length was unchanged between nitrogen treatments. No consistent changes in root hair length or density occurred for root hairs along the crown roots in response to the -N treatment **(D)**. However, in both N treatments, root hairs were generally longer and denser at the top of the crown roots than at the bottom of the crown roots **(D)**.

The growth and acclimation of FM roots and root hairs in response to nutrient stress have not previously been reported. In this study, we have surveyed a range of responses employed by FM for growth in N-poor environments. Consistent with other cereal crop plants (Hay and Porter, 2006), FM shoot, root, and seed head biomass decreased in the -N treatment, associated with declines in shoot tiller number, crown root number, total crown root length and total lateral root length, but with no consistent changes in root hair traits. These results are summarized in a model (**Figure 6**).

### Finger Millet may have Efficient Nitrogen Uptake Ability

The baked-clay gravel (Turface^®^) in which plants were grown intrinsically contains very low N (Supplementary Table S2). The clay gravel used contained 0.053% total N, which is substantially low compared to the surface layer of most cultivated soils in which N content can range between 0.06 and 0.5% (Bremner, 1996; Johnston-Monje et al., 2014). Also, it is important to note that the media utilized in these experiments was not soil, but hard, inert gravel, from which the majority of nitrogen would be unavailable for uptake during the life cycle of the FM plants. Total nitrogen was measured by soaking the clay gravel in the N-free nutrient solution used in the experiments, and was found to be 1.42 mg/L, or 0.1 mM total N. In rice and maize, these conditions would be considered to cause N starvation (Schluter et al., 2012; Yang et al., 2015). To provide perspective, the United States Environmental Protection Agency defines clean drinking water for humans as containing no more than 10 mg/L of N as nitrate – a level more than seven times that which was available to FM plants over the course of our study^2^. Furthermore, our N values are likely overestimates, as they do not take into account leaching of N, given that the coarse clay had a low surface area and hence poor N binding capacity, with large air pockets to facilitate rapid downward nutrient flow away from the rhizosphere through holes in the sides of the pails.

The question then arises as to how FM was able to reach maturity with no deliberately added N, and with such low levels of N endogenous to the growth system. Furthermore, surprisingly, plants showed inconsistent differences in chlorophyll between the -N vs. +N treatments (**Table 3**). In 2013 the plants receiving the -N treatment contained significantly less chlorophyll than the +N plants. This significant difference was not observed in 2012, although the trend remained similar. Additionally, there were no significant differences in tissue N concentration (**Table 3**). In other cereal crops, N starvation has been shown to cause consistent declines in chlorophyll (Széles et al., 2012; Muñoz-Huerta et al., 2013; Wang et al., 2014) and substantial declines in tissue N concentration (Havlin et al., 1999; Chen et al., 2015). Here, the plants were grown on an outdoor field in open pails (**Figures 1B,C**), closed at the bottom but perforated with four drainage holes on the side, very close to the bottom edge (Supplementary Figure S2). We did sometimes observe roots growing from these drainage holes, but only in older plants. It is possible that N was taken up by these late stage roots to assist with grain fill, but nevertheless, the plants would have needed to grow for months without these hypothetical scavenging roots, using starter N from an extremely small seed. Furthermore, we did not observe weeds in the pails (e.g., that might host N-fixing microbes) nor an obvious mycorrhizal network at root harvesting and during root imaging. Some N may have entered from unintended sources such as from animal activity (we observed bird feathers), N-fixation by lightning, algae (growth was observed in pots), or microbial growth in the fertigation lines. These external N inputs were likely miniscule and suggest that FM roots themselves may have excellent N uptake ability.

In the future, it may be worth investigating whether FM hosts N-fixing microbes, as is observed in its grass relative, sugarcane (Cavalcante and Dobereiner, 1988; James et al., 1994). We conducted a preliminary experiment to test whether culturable microbes from FM root and shoot extracts can grow on agar without N, but no colonies appeared (data not shown).

These results also provide further impetus to examine FM as a source of novel agronomic traits for genetic dissection at the molecular level, especially with regards to N uptake and utilization efficiency mechanisms. For example, the behavior of Dof1 and Dof2, transcription factors implicated in controlling many responses to N, have already been characterized in FM under conditions of varying N applications (Gupta et al., 2014, 2015; Kumar et al., 2014). However, other molecular genetic studies concerning N in FM are scarce.

### In the –N Treatment, Shoot, and Total Seed Head Biomass Decreases were Associated with Declines in Tiller Number

Consistent with other cereal crop plants (Hay and Porter, 2006), FM shoot and total seed head biomass decreased in the –N treatment (**Table 1**), which were associated with declines in tiller number in both 2012 and 2013. Inadequate N supply has long been known to lower biomass and yield in plants that display tiller plasticity including wheat, barley, rice, several bioenergy grasses, and teosinte, the progenitor to modern maize (Mae, 1997; Gaudin et al., 2011b; Alzueta et al., 2012; Waramit et al., 2014).

### In the –N Treatment in 2013, Root Biomass Declines were Associated with Diminished Root Traits

In other cereals including maize, low N availability was shown to increase the length of the root system, decrease the crown root number, and increase the lateral root to crown root ratio (Eghball and Maranville, 1993; Wang et al., 2004; Chun et al., 2005; Liu et al., 2009; Gaudin et al., 2011a). However, in FM plants harvested in 2013, we observed a decrease in the total length of both the lateral root system and crown root system in the absence of N fertilizer, as well as a decrease in crown root number (**Table 2**). Lateral root to crown root ratios remained statistically equivalent between treatments. In 2012 root architecture did not differ significantly between treatments.

Differences between our observations and the literature might be explained by the fact that the earlier studies using other cereals were conducted under conditions of N limitation, while our FM plants may have experienced extreme N stress (i.e., 0.1 mM N from the clay growth medium). The majority of experiments thus far investigating plant root responses to true N starvation have been conducted in *Arabidopsis*. A group of *Arabidopsis* ecotypes has been identified which respond to N starvation with decreases in various root traits including root fresh biomass (Ikram et al., 2012). However, it should be noted that the majority of these ecotypes responded by increasing root biomass, indicating there is a substantial degree of genetic diversity in plant root acclimation responses. Given these prior results, it will be helpful to phenotype the roots of multiple genotypes of FM in response to extreme N stress.

Research into the responses of crop plants to N starvation, including in maize and barley, is primarily restricted to molecular analysis, with a focus on the regulation of N assimilation genes (Lee et al., 1992; Møller et al., 2011; Nacry et al., 2013). Additionally, because of the difficulties of imposing true N starvation on nutrient-intensive crops, the stressful condition is often applied only for a short time, unlike the current study in which it was the entire duration of the FM life cycle.

### Finger Millet Displays Whole-Plant Coordination of Architectural Traits in Response to Low N

Tiller number and crown root number were found to be correlated across the N treatments (**Figure 4**), indicating that FM’s above ground and below ground morphologies may be tightly linked. To ensure that this relationship is robust, similar correlations should be generated in the future with additional N treatments (see below). In modern maize, the transcription factor Teosinte Branched 1 (TB1) is in part responsible for the repression of tiller outgrowth, and results in the plant’s single-stem growth habit (Doebley et al., 1995; Lukens and Doebley, 2001). It has been shown that selection pressure during the domestication of maize from teosinte led to changes in expression of the *Tb1* gene and coordinated declines in tiller and crown root number (Hubbard et al., 2002; Gaudin et al., 2011b, 2014). As FM is genetically related to maize, a similar mechanism may be directing the tight correlation between the number of tillers and crown roots and it may be of interest to investigate the presence of *Tb1* orthologs in the FM genome.

### Crown Root Initiation may be the Primary Determinant of Changes in Root Architecture to Support Altered Shoot Growth

Although the relationship between FM yield and above ground traits including plant height and shoot biomass has been previously investigated (Wolie and Dessalegn, 2011), this study is, to the best of our knowledge, the first time that such correlations have been performed on below ground plant architecture in FM. The highest correlations observed between FM shoot and seed head biomass and root traits were with the crown root number, not the average crown root length (**Figure 4**). Furthermore, of the various root traits that changed in response to the -N treatment, the crown root number showed the greatest decline (-49%). Total crown root length was found to correlate tightly with crown root number (**Figure 4G**). Since lateral roots originate from crown roots, then combined these results indicate that declines in crown root initiation in response to N limitation primarily determine the other root correlations observed (**Figure 4**). It seems logical that when plants have increased access to N, they respond by initiating additional crown root systems (with their lateral roots and root hairs) in order to support increased shoot biomass and seed head production.

The correlations generated here may aid future breeding efforts and trait association studies, in which below ground contributors to yield and biomass could be explored. However, a relatively small amount of data was used to generate these correlations, and a degree of prudence must be used in the interpretation of the results. In particular, the consistency of responses within our +N and -N treatments resulted in clustered data (**Figure 4**). In future experiments, it may be useful to add additional N treatments to make the correlation analyses more robust.

As more of the FM genome sequence becomes available it may be helpful to investigate orthologs of genes implicated in crown root initiation in other cereals including *Tb1* (noted above) and *RTCS* identified in maize (Hubbard et al., 2002; Taramino et al., 2007), as well as *Crown rootless 1, 4, 5* and *WOX11* identified in rice (Inukai et al., 2005; Zhao et al., 2009; Coudert et al., 2010; Kitomi et al., 2011). Additional candidate genetic elements include those responsive to auxin or cytokinin signaling, pathways recognized as contributing to the control of crown root growth and development (Inukai et al., 2005; Coudert et al., 2010; Kitomi et al., 2011).

### Low N did not Induce Consistent Changes in Root Hair Length or Density

Based on hand tracing of 14,420 root hairs in this study, we observed no consistent differences in root hair length and density in FM in response to the -N treatment (**Figure 5**; Supplementary Figure S1). In the literature, the analysis of root hairs in response to N limitation/starvation in crop plants is understudied, with contradictory results. Root hairs in *Arabidopsis* and several grass species (*Holcus lanatus* L., *Deschampsia flexuosa* L., *Poa annua* L., and *Lolium perenne* L.) showed increased length and density in response to N limitation (Robinson and Rorison, 1987; Bloch et al., 2011). Root hairs were hypothesized to contain a mechanism for sensing low N stress (Shin et al., 2005). In maize and teosinte, however, root hairs showed declines in length and density as a result of low N conditions using an aeroponics misting system (Gaudin et al., 2011a,b). One potentially interesting observation from the present study is that FM root hairs may have been longer and more dense in response to low N at the tips of some root age classes (**Figures 5C,D**; Supplementary Figure S1A). In the future, precise examination of root hair initiation may help to confirm these observations.

### Study Limitations

To the best of our knowledge, this study is the first attempt to grow FM in the northern latitudes of Canada. A growing season which is cooler and shorter than that of FM’s native East Africa and India (Goron and Raizada, 2015) created a set of challenges and limitations. For example, it was necessary to delay germination and transplantation in the spring until local temperatures could sustain growth. This resulted in important differences between the 2012 and 2013 growing seasons. Although the total time between transplantation and harvest was similar each year (142 DAT in 2012 vs. 139 DAT in 2013), in 2012, transplantation was performed 18 days later and growth extended into November which borders the cold, winter season. In 2012, the late-season conditions became especially sub-optimal for FM which requires average daily minimum temperatures to be above 18°C for optimum growth^3^; the average temperature on the day of harvest was -3.3°C^4^. Plants were stunted with a greatly reduced tiller number in 2012 compared to the 2013 plants (**Table 1**), and as a result the plants were from a different growth stage at the time of harvest (**Figures 2B,D**). Indeed, many biomass measurements (**Tables 1** and **2**) were significantly lower in 2012 than 2013, although the trends remained similar in terms of the response to N treatments. Lower biomass in 2012 may also have been the result of pathogenesis as disease-like spots were observed at the seedling stage (they completely disappeared within a few weeks); these symptoms were absent in 2013. As FM is an indeterminate plant, the early onset of cold in 2012 (compared to 2013) combined with the slow growth of seedlings (perhaps due to the disease) might have prevented the initiation of new tillers and roots in 2012. The cold likely also affected seed heads (**Table 1**), as both grain fill and vegetative growth occur in parallel in FM. Finally, these differences in growing conditions, combined with differences when measurements were taken relative to the growth stage between years, may have also contributed to the inconsistent chlorophyll readings discussed above.

In 2012, the decrease in root biomass from 230 to 56 g in the -N treatment was not matched by large differences in the architectural traits (**Table 2**). In 2012, we were especially cautious while washing the root systems after harvest to preserve root hairs and lateral roots. It is likely that fine Turface^®^ clay stuck to the roots as a result, especially on the larger +N root systems which were more dense and difficult to wash, resulting in a non-linear increase in the biomass. In 2013, the roots were washed more thoroughly.

Another study constraint was that, although the pails used to grow plants were very large, they may have limited the growth of the FM root systems, especially in the +N treatment, thus reducing the significance of the differences between N treatments. A future experiment involving growing plants directly in field soil could be informative, but it would not be possible to impose extreme N stress conditions and would be especially challenging to phenotype fine root traits.

A final study limitation was that in 2012, shoot dry weight was measured while in 2013 shoot fresh weight was measured, creating a challenge to directly compare biomass results between years (**Table 1**). As the leaf tissue in grass species is mostly water (Garnier, 1992; Garnier and Laurent, 1994), the differences between the shoot biomass data in 2012 vs. 2013 are not unreasonable. In any case, all comparisons were made within years to circumvent this issue. For seed heads and roots, however, consistent measurements were used across both years.

### Study Implications and Future Experiments

This study has provided the first detailed description of FM responses to extreme N stress, including the first description of root acclimation responses. The results suggest that FM roots may have extreme N uptake ability. Mapping of the underlying genes may aid marker assisted selection (MAS) based efforts to improve NUE in other cereal crops.

Only a single genotype was examined in this study; however, there are many landraces available for FM from Africa and South Asia that show diverse adaptations to local climates (Jarvis et al., 2008; Wolie and Dessalegn, 2011; Goron and Raizada, 2015). It will be useful to compare the results from this study to a diversity panel of FM to identify those with particularly high NUE and uptake ability. Through such guided breeding approaches, it may be possible to help subsistence farmers in Africa and South Asia who have poor access to N fertilizer.

## Author Contributions

TLG undertook all lab experiments, the 2013 field experiments, performed all analyses and wrote the manuscript. VKB helped to conceive the study, and designed and set up the field experiment in 2012. CRS helped to set up the field experiments and assisted with plant growth and measurements. SW assisted with root hair microscopy and morphometric measurements. MNR conceived of the study and edited the manuscript.

## Conflict of Interest Statement

The authors declare that the research was conducted in the absence of any commercial or financial relationships that could be construed as a potential conflict of interest.

## Footnotes

1 http://smallmillets.res.in/html/reports.html

2 http://water.epa.gov/drink/contaminants/basicinformation/nitrate.cfm#four

3 http://www.cabi.org/cpc/datasheet/20674#tab1-nav

4 http://climate.weather.gc.ca/
