# Supplementary material for: Whole plant acclimation responses by finger millet to low nitrogen stress
Source: Front Plant Sci. 2015 Aug 19;6:652. doi: 10.3389/fpls.2015.00652 (PMC4541148; doi:10.3389/fpls.2015.00652)
Supplement: Supplementary file 1 [file Data_Sheet_1.PDF]

Supplementary Material

Whole plant acclimation responses by finger millet to low nitrogen stress

Travis L. Goron[1], Vijay K. Bhosekar[1], Charles Shearer[1], Sophia Watts[1] and Manish N. Raizada[1]*

[1]University of Guelph, Department of Plant Agriculture, Guelph, ON, Canada

[*]**Correspondence:** Manish N. Raizada, University of Guelph**,** Department of Plant Agriculture**,** 50 Stone Road East**,** Guelph, ON, N1G 2W1, Canada
Fax: 1-519-763-8933
Phone: 1-519-824-4120 x53396
raizada@uoguelph.ca

1. Supplementary Figures and Tables

1.1. Supplementary Tables

Supplementary Table 1. Chemical description of Turface® MVP clay gravel[1].

Chemical property	Description
SiO_2 content	74%
Al_2O_3 content	11%
Fe_2O_3	5%
Content of CaO, MgO, K_2O, Na_2O, TiO_2, and other chemicals	<5%
Cationic exchange capacity (CEC)	33.6 mEq/100g
pH range	5.5 + 1.0
Porosity	Total 74%: 39% capillary, 35% non-capillary

[1] Adapted from Turface® MVP product information sheet (www.turface.com)

Supplementary Table 2. Potential N availability of Turface® MVP clay gravel. Turface® MVP clay gravel was submerged for 24 hours with modified N-free Hoagland's nutrient solution, and the total N content of the resulting solution was determined with the Kjeldahl method. Turface® MVP clay gravel was ground to a fine dry powder and total N content was determined by Dumas combustion. N=3.

	Mean total N content ± SEM
N-free nutrient solution, after soaking with Turface®	1.42 mg/L ± 0.028
Turface® MVP clay gravel	0.053% ± 0.0033

1.2. Supplementary Figures

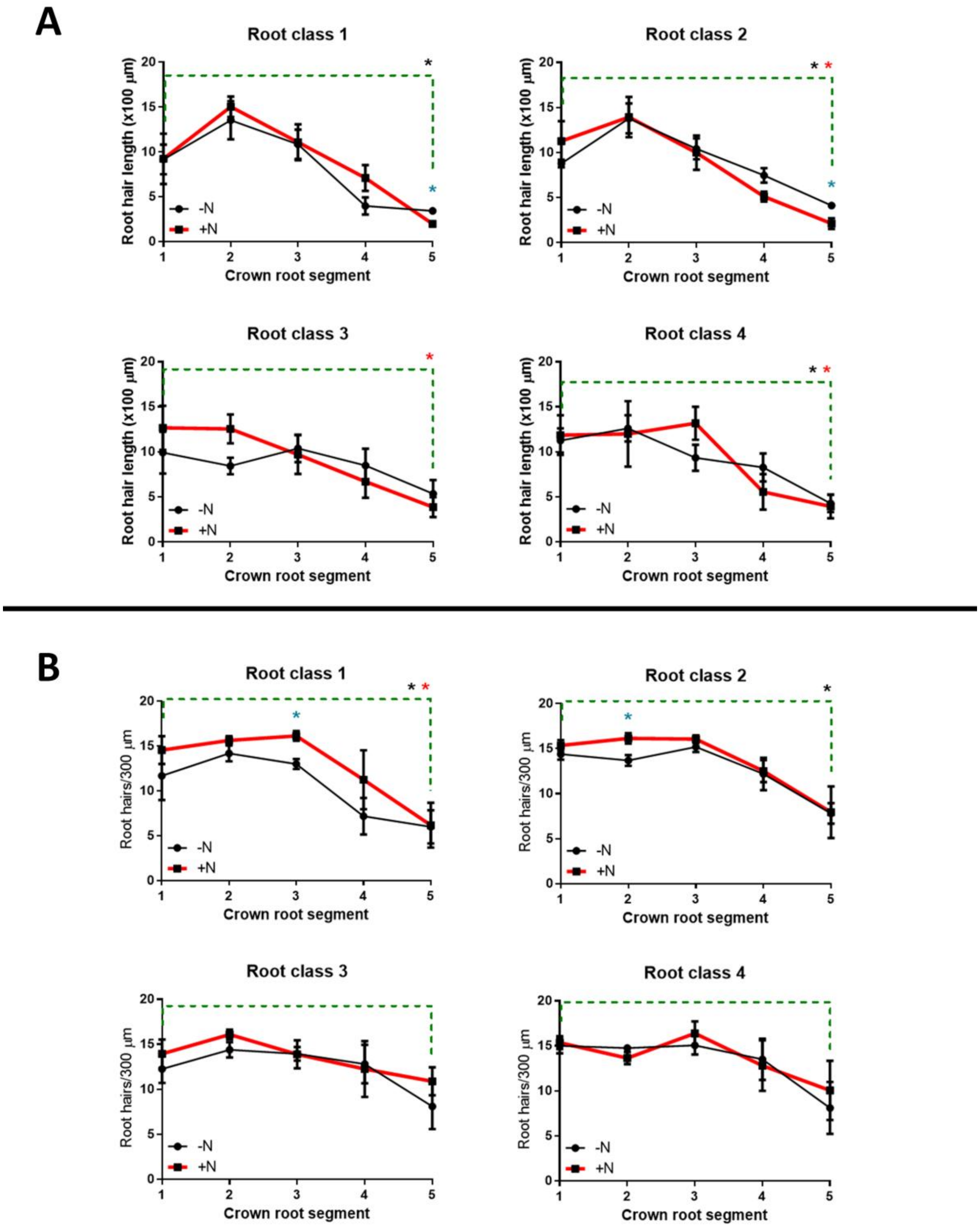

Supplementary Figure 1. Root hair morphometric traits from 2012 plants at harvest. Similar to the 2013 data (**Figure 5**), four different classes of crown roots were sampled based on their

lengths/ages, and then examined for root hairs at five different segments spaced evenly along each crown root using light microscopy. Root hairs were quantified for length (**A**) and density (**B**) at different segments of the crown roots (x-axis) ranging from the top/nearest the shoot (crown root segment 1) to near the root tip (crown root segment 5). A blue asterisk (*) directly above a mean data point denotes a significant difference in the root hair trait (at $p<0.05$) between N treatments within an individual crown root segment. An asterisk above the green dashed line denotes a significant difference in the root hair trait (at $p<0.05$) between the top crown root segment (segment 1) and the bottom crown root segment (segment 2) within the +N treatment (red asterisk) or the –N treatment (black asterisk). All statistical analyses were performed with unpaired t tests (with Welch's correction where unequal variances required).

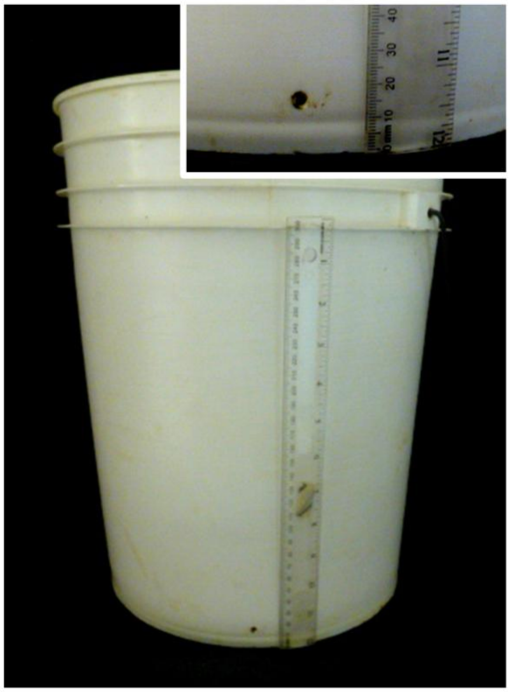

Supplementary Figure 2. Picture of the type of pail used in this experiment, showing the location of one of four perforations on the side. The inset shows an enlarged view.
